# Treatment-related acute toxicity with adjuvant systemic treatment among patients with HER2-positive early invasive breast cancer: a national population-based cohort study

**DOI:** 10.1136/bmjonc-2023-000081

**Published:** 2023-09-04

**Authors:** Melissa Ruth Gannon, David Dodwell, Katie Miller, Jibby Medina, Karen Clements, Kieran Horgan, Min Hae Park, David Alan Cromwell

**Affiliations:** 1 Department of Health Services Research & Policy, London School of Hygiene & Tropical Medicine, London, UK; 2 Clinical Effectiveness Unit, The Royal College of Surgeons of England, London, UK; 3 Nuffield Department of Population Health, University of Oxford, Oxford, UK; 4 National Cancer Registration and Analysis Service, NHS England, Birmingham, UK; 5 Department of Breast Surgery, St James's University Hospital, Leeds, UK

**Keywords:** breast cancer (female), adverse effects, adjuvant therapy

## Abstract

**Objective:**

Although adjuvant trastuzumab-based treatment (TBT) improves survival for patients with HER2-positive early invasive breast cancer (EIBC), risk of toxicity grows as patient age increases. We examined use of TBT and associated severe acute toxicity event (SATE) rates to understand the real-world impact.

**Methods and analysis:**

Women (50+ years), newly diagnosed with HER2-positive EIBC in England, 2014–2019, were identified from Cancer Registry data, linked to the Systemic Anti-Cancer Therapy dataset for TBT information. SATEs were measured using hospital administrative data. Statistical models were developed to identify potential predictors of SATE.

**Results:**

Among 5087 women who received trastuzumab, with median duration 11.7 months, 47.4% (95% CI 46.0% to 48.7%) completed treatment. Women aged 70+ years made up 20.2% of patients aged 50+ who received adjuvant TBT in routine care, compared with 5% of women aged 50+ across trials. 32.8% (95% CI 31.5% to 34.1%) had a record of any SATE. 6.8% (95% CI 6.1% to 7.5%) had a cardiovascular SATE. Congestive cardiac failure rate was 0.5% (95% CI 0.3% to 0.7%). High deprivation, anthracycline use, increasing frailty were associated with increased odds of any SATE. Older age, sequential chemotherapy, history of myocardial infarction/chronic pulmonary disorder/liver disease were associated with increased odds of cardiovascular SATE. Among two-thirds of women not eligible for trial cohorts SATE rates were lower than for trial-eligible patients, explained by baseline differences in patients.

**Conclusion:**

Evidence of treatment-related SATE among patients treated in routine care is needed to inform treatment decisions and counsel older patients. This study provides information on SATE rates for adjuvant TBT, and common types, overall and by age for such discussions.

**WHAT IS ALREADY KNOWN ON THIS TOPIC**

- Adjuvant trastuzumab-based treatment, given as a targeted therapy for HER2-positve early invasive breast cancer (EIBC), is associated with increased cardiotoxicity risk.

**WHAT THIS STUDY ADDS**

- This study is the first comprehensive presentation of severe acute toxicity event (SATE) rates with adjuvant trastuzumab-based treatment for HER2-positive EIBC, with comparisons across patient subgroups defined by age and patient fitness. Data for 5087 women diagnosed from 2014 to 2019 highlighted one-third had a record of any SATE, while around 1 in 15 had a record of a cardiovascular SATE with increased odds as patient age increased.

**HOW THIS STUDY MIGHT AFFECT RESEARCH, PRACTICE OR POLICY**

- Current evidence of treatment-related toxicity in older patients treated in routine care is vital in informing clinical practice by providing reference of average SATE rates, broken down by patient subgroups including age. This information is valuable for discussions around treatment decisions and helping counsel older patients on the side effects of treatment.

## Introduction

Patients with HER2-positive breast cancer had a poor prognosis until the development of effective HER2-directed therapy, shown in randomised controlled trials (RCTs) to improve survival.^1^ Guidelines recommend adjuvant trastuzumab in combination with surgery, chemotherapy and radiotherapy, for patients with HER2-positive early invasive breast cancer (EIBC).^2–5^

While improving survival, adjuvant trastuzumab is associated with increased cardiotoxicity risk, particularly among older patients.^6–8^ Older age was reported to be associated with an increased frequency of cardiac events, notably congestive cardiac failure (CCF) but trial evidence has been limited by the under-representation of older patients.^7–9^ Patients aged 70+ years accounted for an estimated 2.5% of all patients in RCTs evaluating adjuvant trastuzumab, although at least one-third of patients diagnosed annually with BC are 70 or older. Observational studies describing toxicity, including cardiotoxicity, among patients who received trastuzumab-based treatment in the USA, Canada and Europe, have been in cohorts of patients diagnosed prior to 2010, or provided limited detail about specific toxicity events by age.^10–23^ Few studies have examined the safety of trastuzumab-based treatment given to older women in routine care, and there is a gap in our understanding of the treatment-related toxicity for this population. This information is valuable in informing treatment decisions and counselling older patients on the side effects of treatment.

This study aimed to characterise the cohort of women (50+ years) with HER2-positive EIBC who received adjuvant trastuzumab-based treatment in routine care in England, and investigate treatment-related severe acute toxicity events (SATEs). The study is reported according to the REporting of studies Conducted using Observational Routinely-collected Data (RECORD) extension to the STrengthening the Reporting of OBservational studies in Epidemiology (STROBE) guidelines.^24^ Although SATEs are only one aspect of toxicity burden, they are of importance to understand in the management of patients as they are often the cause of early treatment discontinuation which is associated with poorer outcomes.^25^

## Materials and methods

### Data source

This population-based cohort study was undertaken as part of the National Audit of Breast Cancer in Older Patients (NABCOP; full details can be accessed via www.nabcop.org.uk). NABCOP received pseudonymised Cancer Registry data for all women aged 50+ years, with a new BC diagnosis between 1 January 2014 and 31 December 2019, and treated within an English National Health Service (NHS) trust.^26^ Data were linked at tumour level to data from Cancer Outcomes and Services Dataset (COSD), Hospital Episode Statistics Admitted Patient Care (HES-APC),^27^ Systemic Anti-Cancer Therapy dataset (SACT),^28^ national Radiotherapy Dataset (RTDS) and at patient level to the Primary Care Prescription Database (PCPD).^29^

### Study population

We identified women diagnosed with HER2-positive EIBC (stages 1–3A; International Classification of Diseases, 10th revisision [ICD-10] diagnosis code C50), who received surgery within 6 months of diagnosis and had a record of receiving adjuvant trastuzumab-based treatment (commenced within 4 months of primary surgery with no prior trastuzumab) in SACT. BC was classified as HER2-positive where HER2 status was reported as either positive or borderline with a positive HER2-FISH (fluorescence in situ hybridisation) or equivalent molecular test result. To aid interpretation of SATE rates among these women, we defined a comparison group of women with HER2-negative EIBC, who had surgery (with no prior chemotherapy) within 6 months of diagnosis and adjuvant chemotherapy (commenced within 4 months of surgery) recorded in SACT.

Only patients with a recorded or calculated treatment window end date prior to 1 April 2021 and complete data on patient fitness, tumour stage, nodal stage and invasive grade were included.

### Study outcome

Episodes of treatment-related SATE were identified from ICD-10 diagnosis codes recorded in diagnosis fields associated with an overnight hospital admission within hospital administrative data (HES-APC) using a coding framework previously validated in patients with colon cancer (online supplemental table A1).^30^ The study limited the period during which an episode of SATE could occur to the time from the start of adjuvant treatment up to 8 weeks (56 days) after the last reported cycle. The definition of SATE covered a wide range of possible events, including haematological disorders, infection, cardiovascular disorders, neutropenic sepsis and gastrointestinal disorder. We also looked specifically at cardiovascular SATEs.

10.1136/bmjonc-2023-000081.supp1 Supplementary data

For the comparison group of patients with HER2-negative EIBC, the measure of SATEs included events captured in overnight admissions that occurred from the first reported cycle of chemotherapy up to 536 days later, which corresponded to the 90th centile of the duration of trastuzumab-based treatment for HER2-positive EIBC (online supplemental figure A1). Follow-up in HES-APC data was available up to 31 March 2021 for the HER2-positive cohort and comparison group. Deaths related to admissions were defined as those events with death recorded as the discharge method in HES-APC.

### Study variables

Information on trastuzumab-based treatment was extracted from SACT. Trastuzumab was identified from records which contained either trastuzumab, Herceptin or trastuzumab biosimilar in the drug name field. Trastuzumab treatment in which the first recorded cycle was after surgery, and either within 4 months of the surgery date or following chemotherapy, was defined as adjuvant. Trastuzumab frequency was defined based on time between consecutive cycles. As the recommended interval between cycles is 3 weeks, cycles with a gap of 4–6 weeks since the previous cycle were labelled ‘delayed’, while a gap of 7 weeks up to 4 months between consecutive cycles was defined as a ‘treatment break’. Cycles given after a gap of more than 4 months were considered to be a different treatment episode. Cycles with a dose of zero recorded, with no associated hospital admission for treatment, were not counted. All sequential cycles were counted, and a complete course of treatment was defined as 18 cycles, or 17 cycles with a duration of at least 51 weeks. Treatment was defined as discontinued where less than 16 cycles were recorded. Administration route was categorised as subcutaneous where at least one cycle had this route recorded. Chemotherapy was categorised as: ‘sequential’ where cycles were administered prior to the first trastuzumab date, with no cycles delivered during the trastuzumab cycles (online supplemental figure A2); and ‘concurrent’ where any cycles were administered either on the same day as trastuzumab or between trastuzumab cycles (including chemotherapy started prior to and continued during trastuzumab). Trastuzumab and chemotherapy recorded in HES-APC was included as a sensitivity analysis to evaluate the impact of treatment not recorded in SACT, as described previously.^31 32^

Primary surgery was defined as either breast-conserving surgery (BCS) or mastectomy which occurred within 6 months of diagnosis, identified from Office of Population Censuses and Surveys procedure codes within HES-APC records. Radiotherapy use was identified based on records within RTDS that occurred during the initial treatment episode following diagnosis. Endocrine therapy use was identified from the PCPD.^29^

Data on baseline patient and tumour characteristics were taken from Cancer Registry/COSD. Hormone receptor (HR)-positive breast cancer was defined where either ER or PR status were recorded as positive. Deprivation was measured using the Index of Multiple Deprivation 2019 rank, derived from the patient’s postcode at diagnosis and assigned to national quintiles of deprivation (most (1) to least (5) deprived). ICD-10 codes recorded in HES-APC within 2 years prior to diagnosis were used to determine comorbidity burden (0, 1, 2+; defined using the Royal College of Surgeons of England Charlson Comorbidity Index—CCI) and frailty (fit, mild frailty, moderate-to-severe frailty; defined using the Secondary Care Administrative Records Frailty—SCARF index).^33^

Patients with HER2-positive EIBC were categorised as ‘trial eligible’ (yes/no) where application of the inclusion/exclusion criteria and recruited age range of RCTs conducted in the adjuvant setting, using the routine data available, flagged them as being ‘eligible’ for at least one trial (online supplemental table A2).

### Statistical analysis

All data preparation and statistical analyses were conducted using Stata V.17.0.

Patient and tumour characteristics of women receiving trastuzumab-based treatment are presented for the overall cohort, as well as by age.

SATE rates are presented for the overall cohort as well as by age, comorbidity burden, sequential/concurrent chemotherapy use, and HER2 status (HER2-positive/HER2-negative). Kaplan-Meier survival curves were used to visually assess differences in time to first SATE from start of adjuvant treatment/trastuzumab cycle. Multilevel mixed-effects logistic regression models were used to identify factors associated with odds of (cardiovascular) SATE and odds of treatment discontinuation. Models included baseline variables (measured at diagnosis) of age (50–59/60–69/70–79/80+), patient fitness (CCI, SCARF index), deprivation and comorbidity flags for history of myocardial infarction (MI), CCF, chronic pulmonary disease (CPD), liver disorder, renal disorder or diabetes, based on those created to calculate the CCI (online supplemental table A3). Additionally, chemotherapy details (sequential use and anthracyclines use) were included as predictors of SATE.

Further comparison of cohort characteristics and SATE rates was done across patient subgroups defined as ‘trial eligible’.

## Results

Among 156 375 women, aged 50 years and over, diagnosed with EIBC in England between 1 January 2014 and 31 December 2019, 9.6% (n=14 936) had HER2-positive tumours. There were 11 584 of these 14 936 patients who had surgery within 6 months of diagnosis, of which 45.0% (n=5215) went on to receive adjuvant trastuzumab-based treatment (not in combination with other HER2-targetting drugs) with no trastuzumab prior to surgery (online supplemental figure A3). A total of 5087 women had a treatment window ending prior to 1 April 2021 and information on patient fitness and tumour characteristics, and constituted the analysed study cohort. There were 13 577 women diagnosed with HER2-negative tumours over the same time period who received adjuvant chemotherapy with a calculated treatment window ending prior to 1 April 2021. Median follow-up of both groups was 61.1 months (IQR: 44.4–76.1).

### Cohort characteristics

Table 1 provides patient and tumour characteristics plus treatment details for the cohort of women receiving adjuvant trastuzumab-based treatment for HER2-positive EIBC. The majority (79.8%) of the cohort was aged 50–69 years, had no comorbidities (90.6%) and were fit (85.7%). Two-thirds had hormone receptor-positive cancers. The distribution of tumour characteristics differed by age, with a greater proportion of older women having higher grade disease, larger tumours and nodal involvement. Comorbidities or some level of frailty were also more prevalent in older age groups. Rates of BCS and radiotherapy were lower for women aged 80+ years. While nearly all women were recorded as receiving chemotherapy, the regimen type differed by age, with older women more likely to receive a taxane-only (paclitaxel use increased as age increased, while docetaxel use decreased).

**Table 1:** Distribution of patient, tumour and treatment characteristics in women with HER2-positive, early invasive breast cancer, diagnosed in NHS trusts in England between January 2014 and December 2019 and receiving adjuvant trastuzumab-based treatment, overall and by age at diagnosis

Total		Total	50–59 years	60–69 years	70+ years
		5087	2153	1906	1028
Age group	50–59 years	2153 (42.3%)			
	60–69 years	1906 (37.5%)			
	70–79 years	941 (18.5%)			
	80+ years	87 (1.7%)			
IMD 2019	1—Most deprived	15.2%	17.5%	13.9%	12.8%
	4	16.7%	17.6%	16.4%	15.7%
	2	21.4%	20.9%	22.3%	20.7%
	3	22.8%	21.6%	23.6%	24.2%
	5—Least deprived	23.8%	22.5%	23.9%	26.6%
Charlson Comorbidity Index	0	90.6%	93.6%	89.2%	86.9%
	1	7.5%	5.3%	8.4%	10.2%
	2+	1.9%	1.0%	2.4%	2.9%
SCARF index	Fit	85.7%	90.8%	84.2%	77.7%
	Mild frailty	9.3%	6.8%	10.1%	13.1%
	Moderate-to-severe frailty	5.0%	2.5%	5.7%	9.1%
Stage grouping	1	46.5%	49.4%	51.7%	30.8%
	2	46.4%	44.3%	43.1%	57.0%
	3A	7.1%	6.3%	5.2%	12.2%
Grade of disease	G1	1.9%	2.6%	1.7%	1.0%
	G2	36.7%	36.9%	39.7%	30.7%
	G3	61.4%	60.5%	58.6%	68.3%
Tumour stage	T1	56.3%	59.7%	60.7%	41.0%
	T2	40.4%	37.3%	37.1%	52.9%
	T3	3.3%	2.9%	2.2%	6.1%
Nodal stage	N0	68.8%	68.8%	73.7%	59.7%
	N1	25.4%	26.1%	21.8%	30.5%
	N2	5.8%	5.1%	4.6%	9.7%
Positive hormone-receptor status	Yes	68.9%	72.5%	68.2%	62.5%
	No/unknown	31.1%	27.5%	31.8%	37.5%
Surgery type	BCS	68.4%	70.5%	72.1%	57.0%
	Mastectomy	31.6%	29.5%	27.9%	43.0%
Radiotherapy reported/setting	No	20.7%	20.3%	19.1%	24.7%
	Yes—before trastuzumab	5.9%	5.2%	7.0%	5.5%
	Yes—during trastuzumab	58.1%	60.3%	59.2%	51.8%
	Yes—after trastuzumab	15.2%	14.3%	14.7%	18.0%
Hormone therapy prescribed (Y)		69.0%	72.4%	68.7%	62.4%
Chemotherapy reported (Y)		98.6%	98.9%	98.8%	97.5%
	Anthracycline* only (Y)	18.2%	16.1%	21.4%	16.4%
	Taxane* only (Y)	41.3%	31.7%	40.7%	62.8%
	Anthracycline and taxane (Y)	39.6%	51.5%	36.8%	19.8%
	Anthracycline prior, taxane concurrent (Y)	37.2%	48.7%	34.9%	17.4%
	Docetaxel (Y)	49.5%	61.6%	45.6%	31.2%
	Paclitaxel (Y)	32.7%	22.9%	33.4%	52.1%
	Sequential chemo (Y)	21.9%	20.0%	24.8%	20.5%
	Concurrent chemo (Y)	78.1%	80.0%	75.2%	79.5%
	Concurrent anthracycline (% of concurrent chemo)	0.6%	0.8%	0.4%	0.5%
	Concurrent taxane (% of concurrent chemo)	98.9%	99.3%	98.8%	98.3%
Trastuzumab discontinued (Y)		34.4%	32.9%	34.6%	37.1%

*Anthracyclines = doxorubicin, epirubicin, mitoxantrone recorded in SACT. Taxanes = docetaxel, cabazitaxel, paclitaxel, nab-paclitaxel recorded in SACT.

BCS, breast-conserving surgery; IMD, Index of Multiple Deprivation; NHS, National Health Service; SACT, Systemic Anti-Cancer Therapy dataset; SCARF, Secondary Care Administrative Records Frailty.

Based on reported eligibility criteria for trials of adjuvant trastuzumab, 35.6% of the study cohort were categorised as trial eligible. Comparison with reported characteristics of patients within RCTs highlighted that women treated in routine practice include a higher per cent of older women (5% of women aged 50+ in the RCTs were aged 70+ years, compared with 20.2% in this cohort), higher percentages with HR-positive, smaller, node-negative tumours and undergoing BCS in contrast to mastectomy.

The cohort diagnosed with HER2-negative EIBC who received adjuvant chemotherapy had a similar distribution across most characteristics with small differences in relation to age, stage grouping, invasive grade, tumour stage, nodal involvement and use of radiotherapy (online supplemental table A4). The groups differed in the type of chemotherapy used, with more use of anthracyclines (with or without taxanes) and less use of paclitaxel in the comparison group of women with HER2-negative EIBC.

### Trastuzumab treatment details

Median duration of trastuzumab (time from first to last recorded cycle) was 11.7 months (IQR=10.4–12.0), 54.1% of patients had 17–18 cycles and 95.8% (n=4873/5087) had a record of trastuzumab ever being administered subcutaneously. In patients who had more than one cycle of trastuzumab, the typical interval was 3 weekly for 96.1% (n=4802/4998), with only 64 patients (1.3%) having weekly treatment (online supplemental figure A4).

Of 5015 patients with a record of adjuvant chemotherapy, 21.9% (n=1099) commenced trastuzumab after chemotherapy (sequential), while 78.1% (n=3916) received concurrent chemotherapy, of which nearly all (98.9%) included a taxane (table 1). Concurrent taxane use was high across all age groups. Trastuzumab was started after an anthracycline and given concurrently with a taxane for 37.2% (n=1867), with highest use among younger women. Across all age groups, <1% received an anthracycline concurrently.

### Severe acute toxicity events

Among women who received adjuvant trastuzumab-based treatment, 32.8% (n=1670; 95% CI 31.5% to 34.1%) had at least one SATE (table 2). The percentage of women with a cardiovascular SATE captured was 6.8% (n=346; 95% CI 6.1% to 7.5%). Rates of CCF captured were low at 0.5% (95% CI 0.3% to 0.7%). Fifteen (0.3%) women were reported to have died during an admission with a SATE.

**Table 2:** Frequency of severe acute toxicity events (SATE), overall/by individual SATE type, among women receiving adjuvant trastuzumab-based treatment for HER2-positive, early invasive breast cancer, by age at diagnosis (ordered by most frequently recorded; only individual SATE with >5% presented)

Event	Total N=5087		50–59 years N=2153		60–69 years N=1906		70–79 years N=941		80+ years N=87	
	N	%	N	%	N	%	N	%	N	%
Any	1670	32.8	710	33.0	625	32.8	312	33.2	23	26.4
Haematological	774	15.2	325	15.1	304	15.9	142	15.1	3	3.4
Neutropenia	714	14.0	302	14.0	287	15.1	124	13.2	1	1.1
Anaemia	103	2.0	33	1.5	38	2.0	30	3.2	2	2.3
Thrombocytopaenia	15	0.3	6	0.3	7	0.4	2	0.2	0	0.0
Infection	773	15.2	333	15.5	289	15.2	140	14.9	11	12.6
Neutropenic sepsis	659	13.0	279	13.0	267	14.0	112	11.9	1	1.1
Gastrointestinal	507	10.0	193	9.0	199	10.4	107	11.4	8	9.2
Cardiovascular	346	6.8	132	6.1	125	6.6	83	8.8	6	6.9
Arrhythmia	106	2.1	43	2.0	36	1.9	23	2.4	4	4.6
Hypertension	73	1.4	26	1.2	29	1.5	18	1.9	0	0.0
Angina	7	0.1	2	0.1	3	0.2	2	0.2	0	0.0
Congestive cardiac failure	25	0.5	2	0.1	12	0.6	9	1.0	2	2.3
Cerebrovascular	14	0.3	6	0.3	3	0.2	4	0.4	1	1.1
Other	175	3.4	65	3.0	72	3.8	37	3.9	1	1.1

Admissions could contain several conditions related to the SATE. Treating these separately, the most common SATEs, captured among more than 10% of patients, were haematological disorder (15.2%; most commonly neutropenia), infection (15.2%), neutropenic sepsis (13.0%), gastrointestinal disorder (10.0%).

**Figure 1:**
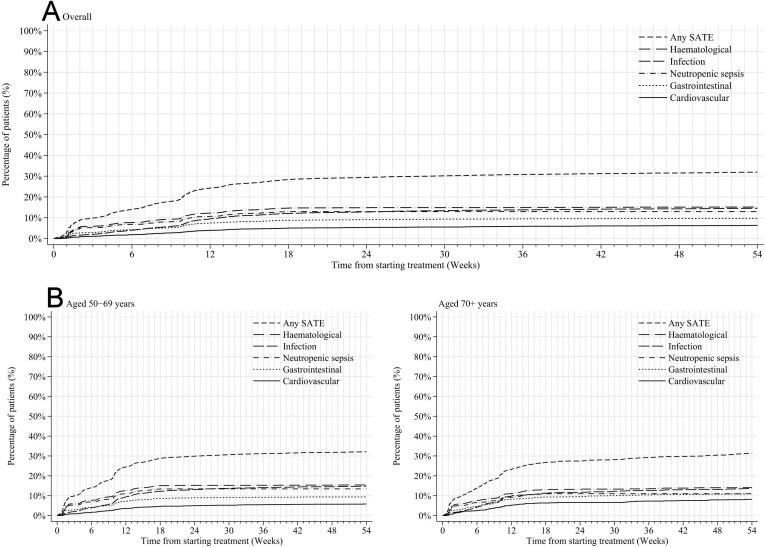
Time from first treatment cycle to first severe acute toxicity event, any and most frequently reported individual toxicity group, among women receiving adjuvant trastuzumab-based treatment for HER2-positive, early invasive breast cancer, (A) overall and (B) by age at diagnosis. SATE, severe acute toxicity event.

**Figure 2:**
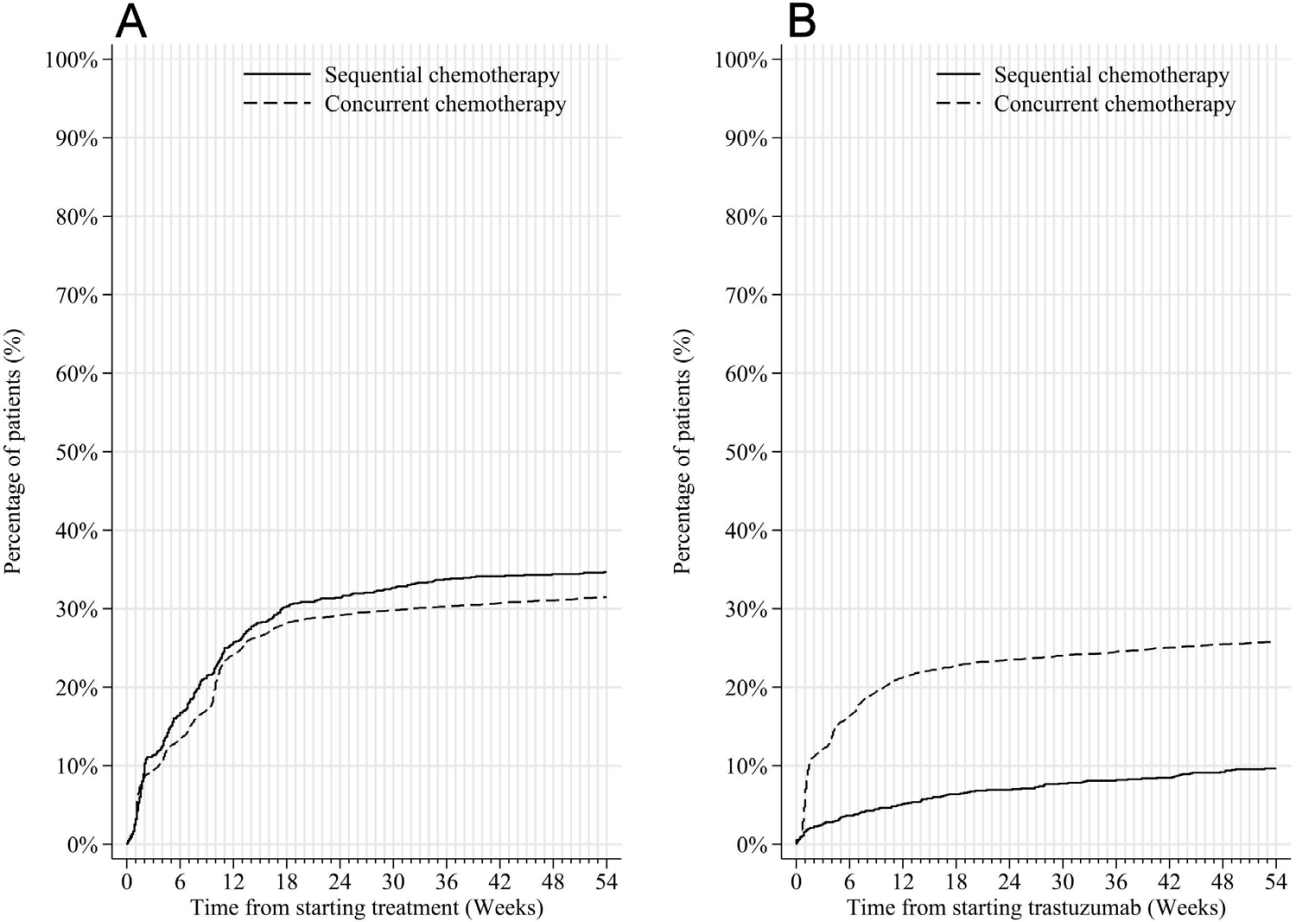
Time to severe acute toxicity event from (A) first treatment cycle and (B) first trastuzumab cycle, among women receiving adjuvant trastuzumab-based treatment for HER2-positive, early invasive breast cancer, by sequential or concurrent use of chemotherapy.

The odds of SATE were greatest during the first 18 weeks of treatment (figure 1). Although there was no difference in SATE rates over the full treatment course according to receipt of sequential versus concurrent chemotherapy (online supplemental table A5), SATEs recorded in overnight admissions after trastuzumab started were higher where patients received concurrent rather than sequential chemotherapy (figure 2). Nearly all concurrent chemotherapy involved a taxane; anthracycline regimens were typically given sequentially. Figure 2 highlights that SATE among patients receiving sequential chemotherapy were lower during trastuzumab suggesting the majority were experienced during chemotherapy rather than trastuzumab. SATE rates did not differ according to whether patients received the full course of trastuzumab treatment or not (33.4% vs 32.3%).

Comparison with the group of women who received chemotherapy for HER2-negative EIBC, overall SATE rates captured were higher for HER2-negative EIBC (37.7%, 95% CI 36.9% to 38.5%; Online supplemental figure A5 and table A6), and cardiovascular SATE rates were also higher (8.5%, 95% CI 8.1% to 9.0%). There were no evidence rates differed after accounting for baseline differences in the two groups. A similar pattern from the first treatment cycle to the first SATE was observed for both groups (online supplemental figure A5).

Overall SATE rates were similar for patients of different ages (online supplemental figure A6). Rates among women aged 80+ were 26.4% (95% CI 17.6% to 37.0%; table 2). SATE rates increased with a greater comorbidity burden, particularly in relation to the SATE due to infection, cardiovascular disorders and gastrointestinal disorders (online supplemental table A7). The rates of cardiovascular disorders rose with increasing age.

High deprivation, use of anthracyclines and greater frailty were associated with increased odds of any SATE, even after accounting for each other and other factors (online supplemental table A8). Older age, history of MI, CPD and liver disease, as well as having sequential chemotherapy, were associated with increased odds of cardiovascular SATE (online supplemental table A8).

### Relationship between SATE and treatment discontinuation/delay

**Figure 3:**
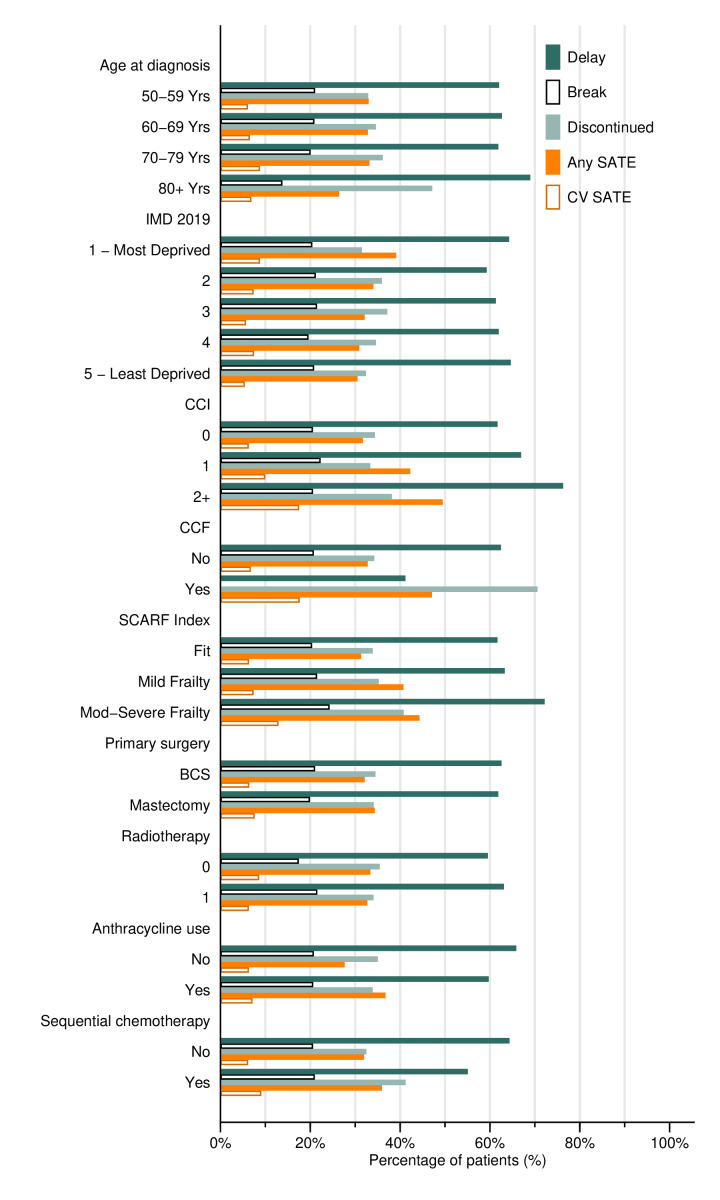
Trastuzumab delays, breaks, discontinuation and SATE rates, among women with HER2-positive, early invasive breast cancer, diagnosed in NHS trusts in England between January 2014 and December 2019 and receiving adjuvant trastuzumab-based treatment, by cohort characteristics. BCS, breast-conserving surgery; CCF, congestive cardiac failure; CCI, Charlson Comorbidity Index; CV, Cardiovascular; IMD, Index of Multiple Deprivation; NHS, National Health Service; SATE, severe acute toxicity event.

Of all women in the cohort, 47.4% (95% CI 46.0% to 48.7%; n=2409/5087) received the complete course of trastuzumab treatment (18 cycles or 17 cycles over at least 51 weeks). A further 18.3% (n=930) had 16 cycles or 17 cycles with less than 51 weeks duration. The remaining 34.4% (n=1748/5087) of women were defined as having discontinued treatment (online supplemental figure A4; table 1). Discontinuation was higher among those receiving sequential chemotherapy (figure 3; 41.2% compared with 31.8% among women receiving concurrent chemotherapy). Additionally, odds of discontinuation were higher among women aged 80+, those with a history of CCF and those who had a treatment break, while having a delay was associated with decreased odds.

Of 4998 patients who received more than one cycle, 69.9% (n=3494) had at least one delay/break between cycles; 63.5% of patients had at least one cycle delayed while 21.1% had at least one break between trastuzumab cycles. Although there was no evidence of a difference by age, the percentage who either discontinued treatment following a SATE (figure 3) or had a delay/break before the next cycle increased with age (online supplemental table A9). Around two-thirds of patients had no SATE captured during trastuzumab cycles but still had a delay/break in between cycles (online supplemental table A9).

### Estimation of SATE rates when the cohort was limited to a ‘trial-eligible population’

Applying the RCT eligibility criteria to our cohort receiving adjuvant trastuzumab-based treatment, 35.6% were defined as ‘trial eligible’. Comparisons of characteristics found lower percentages of trial eligible patients among patient groups of moderate-to-severe frailty, with greater comorbidity burden, grade 1 disease, smaller, node-negative tumours, among women having BCS and by chemotherapy type (online supplemental table A10).

Higher rates of overall SATE were seen among those defined as ‘trial eligible’ compared with those not eligible (36.8% vs 30.6%, p<0.001). This difference was greatest among the group of patients who received concurrent chemotherapy (38.9% vs 30.0%, p<0.001). There were no evidence rates differed after accounting for baseline differences in the two groups.

## Discussion

This study examined the characteristics and treatment safety profile of 5087 women (50+ years) with HER2-positive EIBC who received adjuvant trastuzumab-based treatment. Median trastuzumab duration was 11.7 months, in line with the expected duration of 12 months. One in three women (32.8%) had at least one SATE captured within an overnight hospital admission throughout the course of their adjuvant trastuzumab-based treatment. SATEs were more likely among women with frailty and those receiving anthracyclines. One in 15 women (6.8%) had a cardiovascular SATE captured, with increased rates among older women, and those with a history of cardiovascular or liver disease. Comparison with the group of women receiving chemotherapy for HER2-negative EIBC highlighted comparable SATE rates, after accounting for differences in the cohorts, suggesting trastuzumab did not add toxicity beyond that experienced from chemotherapy.

Comparison with RCTs highlighted differences in cohort characteristics, with women (50+ years) with HER2-positive EIBC treated in routine care, nearly all receiving 3-weekly trastuzumab, including a higher percentage of older women (20.2% aged 70+, compared with 5% of patients aged 50+in the RCTs) and small, node-negative tumours.

Reviews of the four major adjuvant RCTs highlighted rates of CCF varied from 0.6% to 3.3%, with overall cardiovascular toxicity ranging from 5.7%–18.0% and higher odds as age increased.^34 35^ Rates of CCF SATE estimated for our study cohort were 0.5%, with overall cardiovascular SATE rates of 6.8%.

Estimates of trastuzumab-based treatment use in this older population are in line with other studies in this setting.^22 23^ Nearly all had trastuzumab administered subcutaneously, in line with more recent advances which demonstrated clinical comparability with this delivery route.^36 37^ Women aged 80+ were more likely to discontinue treatment, as reported in a previous population-based study in the USA.^38^ Cardiotoxicity and CCF rates reported in other population-based studies provide a mixed picture, with some studies reporting very low rates from 2.6% to 8.5%^14–17^ while others report higher rates up to 29.4%.^13 18 39^ This variation is likely to be due to differences in patient cohorts and data collection methods. Cardiotoxicity captured in hospital admissions data were lower than those reported across several studies, while CCF rates were similarly low: a Dutch study in a hospital setting, which defined cardiotoxicity using the same definition as the HERA trial reported 12.6% cardiotoxicity; a US study in older patients (66+ years) reported 1.2% admissions for CCF; the OHERA study reported 1.0% severe CCF and 17.5% cardiac events; a meta-analysis of studies reported 12% overall cardiotoxicity incidence; and a study across three NHS trusts reported 15.7% cardiotoxicity rates during treatment.^10 12 38 40 41^ Several studies have reported age differences in cardiotoxicity, with risk increasing with age.^12–16^ Additionally, sequential therapy use has been described in another population-based study to be associated with increased odds of cardiovascular SATEs.^42^ Prevalent hypertension captured in the data was low; however, recent publications highlight the risk of chemotherapy-induced hypertension.^43 44^

The study has a number of strengths. It used a large, population-based sample, which included women diagnosed over a period of 6 years (2014–2019) with HES-APC data to 31 March 2021 and so reflects current treatment practice. All data used in the study were linked at patient or tumour level, so all estimates of treatment characteristics and SATE are for the same patients. The cohort in this study included more patients aged 70+ years than were included in the RCTs. The methodology used to identify systemic treatment-related SATEs incorporates toxicity recorded in all diagnosis fields within hospital administrative data and will, therefore, document both those severe events causing an overnight admission as well as symptomatic events experienced and flagged during an overnight admission but which were not the cause for the admission. Pre-existing comorbidities were also accounted for to avoid misclassification of chronic conditions as toxicity, providing more certainty that SATEs were treatment related. Finally, the dataset contained longitudinal treatment information, along with sufficient patient and tumour characteristics associated with treatment decisions to provide robust comparison with trial populations.

There are various limitations of this study. First, as estimation of treatment-related toxicity is based on an overnight stay captured in hospital admissions data, treatment-related toxicity which is either purely symptomatic/less severe or identified via purposeful clinical observation and does not result in an overnight NHS hospital admission, or is managed in a non-NHS setting will not be counted. For this study, many of the toxicities captured will, therefore, predominantly relate to more severe events, the type of toxicity a trial would classify as a serious adverse event. This will result in an under estimate of the true toxicity burden of treatment and as such whether cardiac toxicity rates are different to those observed in the RCTs, which use a different method of measurement to that applied in this study, is unclear. Furthermore, it was not possible within the routine data to know whether the SATE was considered to be a reaction to treatment (serious adverse reaction), something which could be recorded by clinicians within an RCT. Additionally, time to SATE will be an overestimate where the SATE is captured within an admission record but was not the reason for admission, as this will have developed prior to the admission. Second, the time frame for chemotherapy is typically substantially shorter than for trastuzumab-based treatment, therefore, a time window for counting treatment-related admissions among women receiving chemotherapy for HER2-negative EIBC may include admissions relating to any treatments given beyond the initial chemotherapy. Third, as this study used routine data, which were not created or collected to answer this specific research question, there may be issues such as misclassification bias, unmeasured confounding and missing data. As SACT provides data on prescribed therapies, there may be a small number of patients included within the study cohort who were prescribed trastuzumab but for whom it was never administered. Additionally, estimates of treatment cycles/duration may be higher than in practice. As this study aimed to compare the patient cohort and SATE rates to those reported in the trials, where information was presented on an intention-to-treat basis so may also include patients randomised to treatment who never received it or who did not have all treatment cycles, the data provided is informative and complementary. SACT also has various quality assurance processes carried out before data release, details of which can be found via: https://digital.nhs.uk/ndrs/data/data-sets/sact. Another concern is the potential for errors in patient and tumour characteristics within the England Cancer Registry and COSD datasets. The cancer registration service has various validation steps when compiling the national registration data and the overall effect of coding errors should therefore be minimal. We note that missing data for this cohort were small; in addition, sensitivity analysis using HES data to identify further treatment information highlighted few patients were missed from the cohort. Finally, it was not possible to perform a comprehensive comparison of the patients treated in routine practice with those recruited to the RCTs. In part, this was due to the limited reporting of baseline characteristics in the RCT publications and because many of the baseline function tests required prior to enrolment are either not routinely done outside of a trial setting or the details are unavailable within routine national data. As such the estimated number of patients fulfilling trial eligibility is likely to differ from reality. Additionally, this study did not have information on whether any patients in the study cohort were participating in RCTs, which might contribute to SATE rates being under-estimated and to some patients not being recorded as receiving 12 months of treatment.^45^

This study estimated SATE rates for patients receiving adjuvant trastuzumab-based treatment for HER2-positive EIBC in routine care. Overall rates were comparable by age, suggesting patients were well monitored, with an increase in cardiotoxicity as age increased, most likely related to an increased susceptibility to cardiovascular problems due to reduced physiological reserve. Few studies have reported overall SATEs, or individual SATEs beyond cardiotoxicity. Reporting the full safety profile of trastuzumab-based treatment is important in understanding the impact of treatment in routine care, and acknowledging that chemotherapy is part of the treatment provided. Detail of SATE is also valuable in providing information for treatment discussions between clinicians and patients. We found that SATEs were higher among women receiving anthracyclines as part of their chemotherapy treatment, which were typically given sequentially. The majority of this cohort treated in routine care received adjuvant chemotherapy, as is recommended practice, however, we note that more recent trials looking at use of trastuzumab monotherapy have suggested that it might be more appropriate for those patients who are more frail or where SATEs are a concern.^46 47^ Future work looking at SATE for patients receiving trastuzumab monotherapy would be beneficial to understand the safety profile of this among patients treated in routine care. Additionally, frailty rather than increasing age was associated with increased SATEs, re-enforcing the message that age alone should not determine treatment decisions.^48^

Since trastuzumab was first approved for use in this setting in 2005, there have been several further HER2-targeting therapies licensed and approved and a move towards use of trastuzumab biosimilars.^49^ Future research should characterise the cohort of patients receiving these newer treatments to understand the associated benefits and harms from their use in routine care.

In conclusion, this national cohort study found that among patients who received adjuvant trastuzumab-based treatment for HER2-positive EIBC in routine clinical practice, one-third had any SATE recorded, with frailty and use of anthracyclines associated with increased odds. Rates of cardiovascular SATE increased with increasing age and use of sequential therapy. CCF rates were low. The addition of trastuzumab to chemotherapy added little to major SATE experienced, suggesting that where chemotherapy is recommended for HER2-positive EIBC trastuzumab should also be recommended. Two-thirds of patients were estimated to not be represented in trial populations; lower SATE rates among such patients were explained by baseline differences in patients.

10.1136/bmjonc-2023-000081.supp2 Supplementary data

## Data Availability

No data are available. This work uses data that have been provided by patients and collected by the NHS as part of their care and support. The data for England are collated, maintained and quality assured by the National Disease Registration Service (NDRS), which is part of NHS England. Data on English Cancer Registrations can be accessed via the NHS Digital Data Access Request Service (DARS) https://digital.nhs.uk/services/data-access-request-service-dars%23national-disease-registration-service-ndrs-.
