# Supplementary material for: Treatment-related acute toxicity with adjuvant systemic treatment among patients with HER2-positive early invasive breast cancer: a national population-based cohort study
Source: BMJ Oncol. 2023 Sep 4;2(1):e000081. doi: 10.1136/bmjonc-2023-000081 (PMC11234988; doi:10.1136/bmjonc-2023-000081)
Supplement: Supplementary data [file bmjonc-2023-000081supp001.pdf]

Gannon et al. BMJ Oncology 2023.

Treatment-related acute toxicity with adjuvant systemic treatment among patients with HER2-positive early invasive breast cancer: A national population-based cohort study.

Supplementary Material

Figure A1: Time window for assessment of severe acute toxicity event.

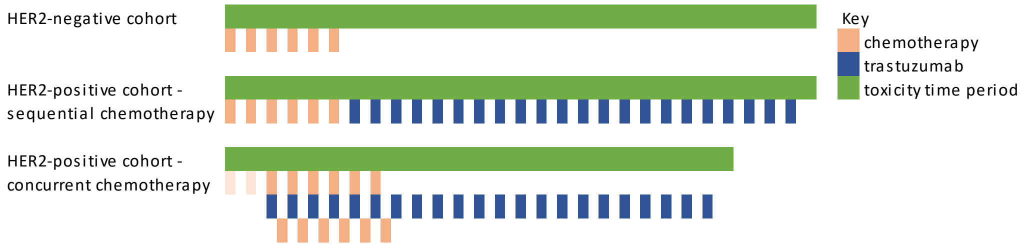

Figure A2: Timing of chemotherapy in relation to commencing adjuvant trastuzumab.

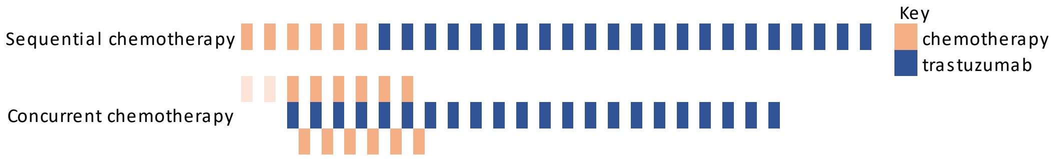

Gannon MR, *et al. BMJ Oncology* 2024; 2:e000081. doi: 10.1136/bmjonc-2023-000081

Figure A3: Details of patient selection from women aged 50 and over, diagnosed with breast cancer in England between 2014 and 2019.

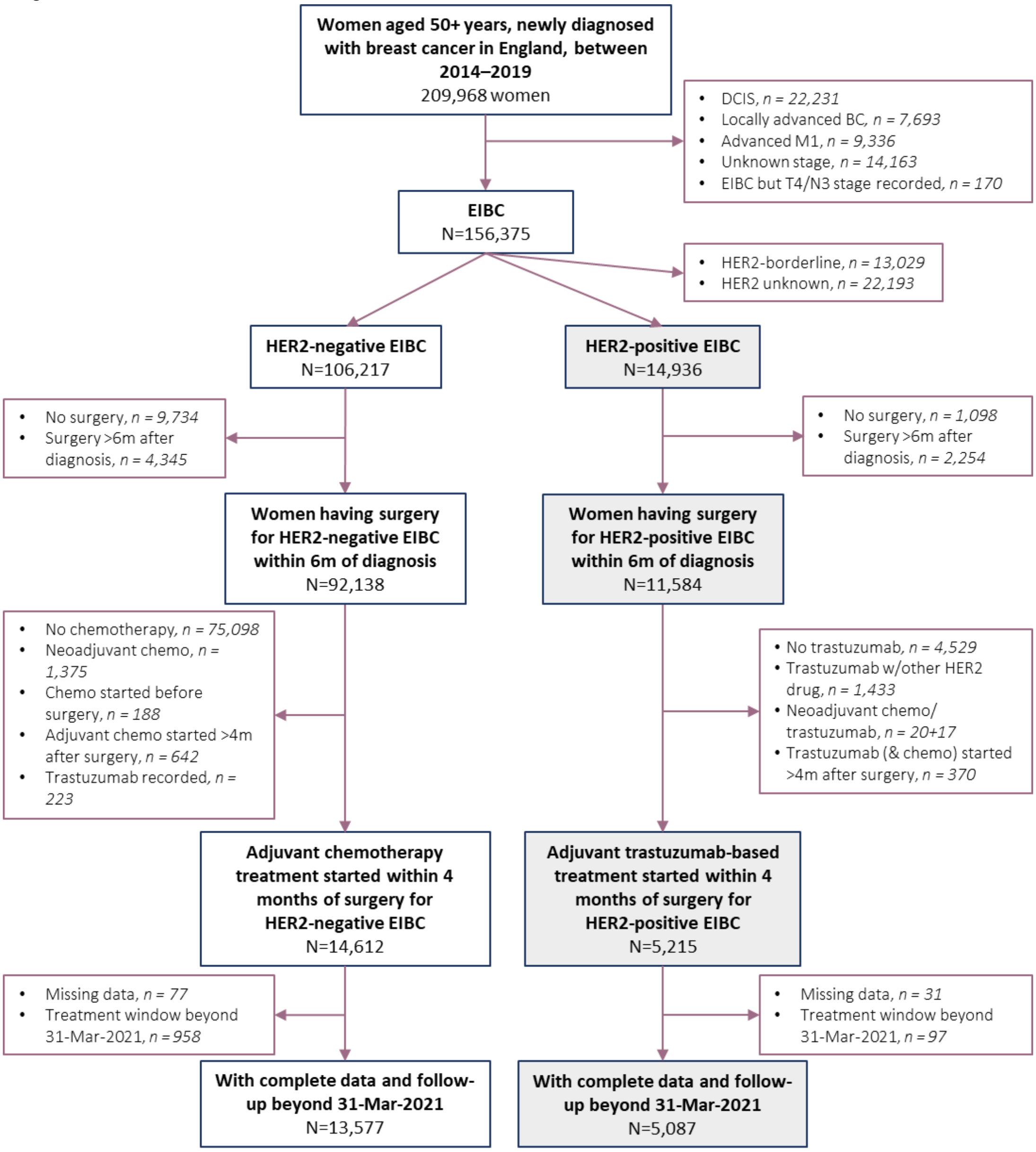

Gannon MR, et al. BMJ Oncology 2024; 2:e000081. doi: 10.1136/bmjonc-2023-000081

Figure A4: Trastuzumab treatment details among women receiving adjuvant trastuzumab-based treatment for HER2-positive, early invasive breast cancer.

Duration of treatment by total number of cycles.

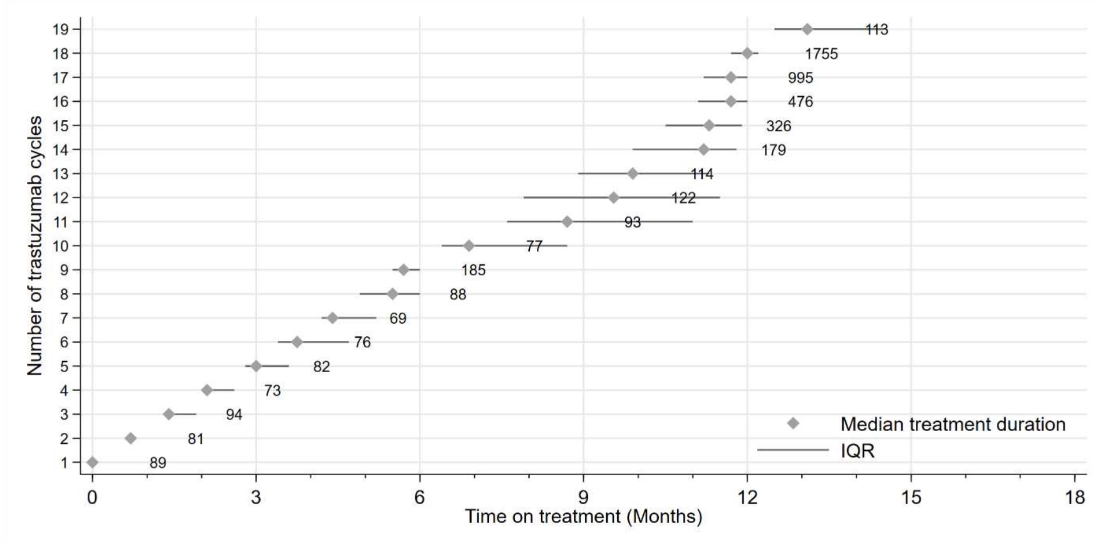

Frequency of trastuzumab cycles and percentage of women receiving each cycle, by cycle number, among women receiving more than one cycle of trastuzumab.

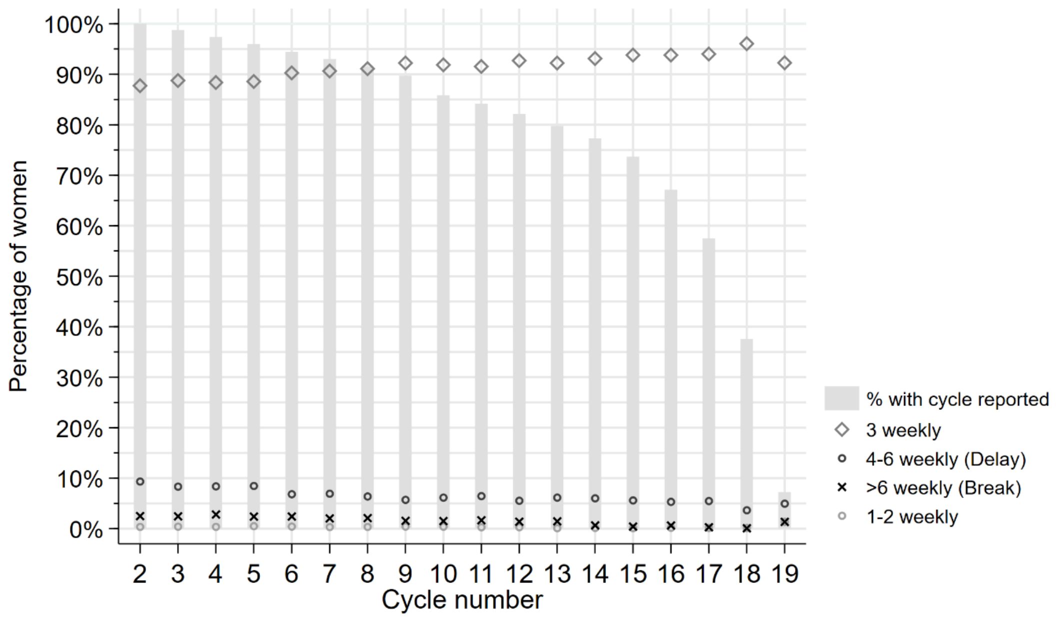

Figure A5. Time from first treatment cycle to first (a) severe acute toxicity event (b) cardiovascular disorder, among women with early invasive breast cancer, by HER2 status.

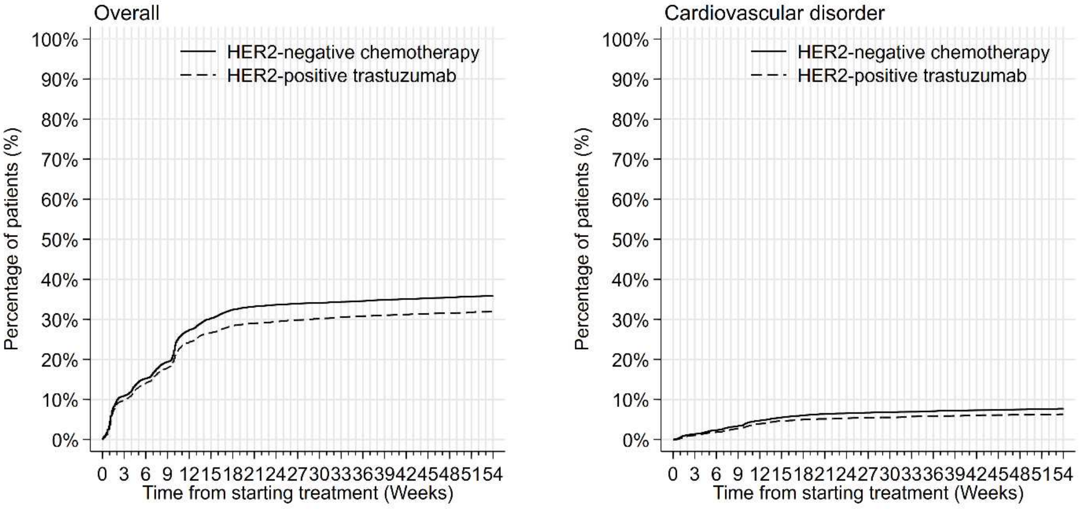

Figure A6: Percentage of women with a severe acute toxicity event, among women receiving adjuvant trastuzumab-based treatment for HER2-positive, early invasive breast cancer, by age at diagnosis

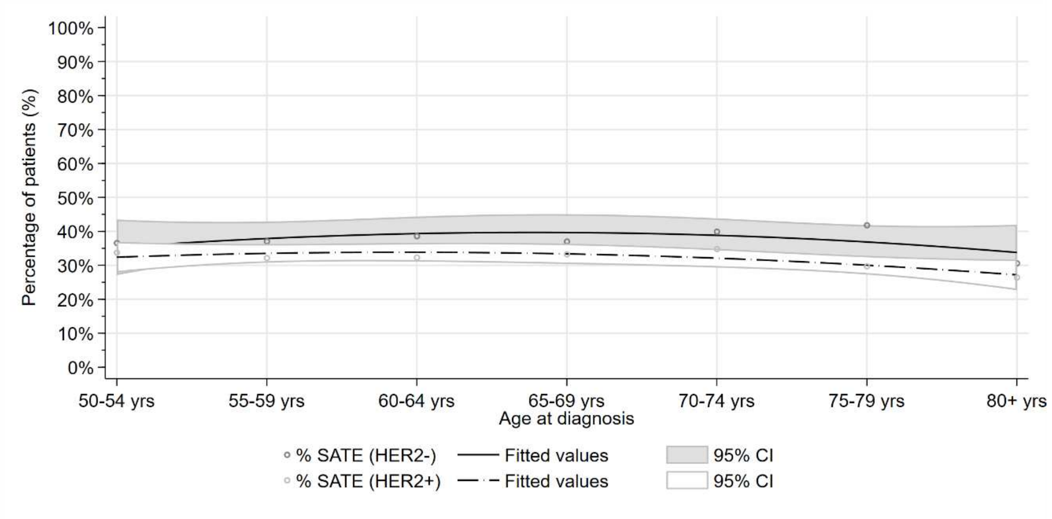

Gannon MR, *et al. BMJ Oncology* 2024; 2:e000081. doi: 10.1136/bmjonc-2023-000081

Table A1: Validated coding framework used to determine severe acute toxicity.

Event	ICD-10 code
**Haematological**	
Neutropenia	D701 D702 D703 D708 D709 D70X
Anaemia	D611 D618 D619 D648 D509* D630 D649*
Thrombocytopenia	D695 D696 D699 M311 R233
**Infection**	R502 R508 R509 R680 R650 R651 R659 A410 A411 A412 A413 A414 A415 A418 A419 A020 A021 A022 A028 A029 A040 A041 A042 A043 A044 A045 A046 A047 A048 A049 A050 A051 A052 A053 A054 A058 A059 A070 A071 A072 A073 A078 A079 A080 A081 A082 A083 A084 A085 A150 A151 A152 A153 A154 A155 A156 A157 A158 A159 A170 A171 A178 A179 A180 A181 A182 A183 A184 A185 A186 A187 A188 A190 A191 A192 A198 A199 A38 A38X A390 A391 A392 A394 A395 A398 A399 A400 A401 A402 A403 A408 A409 A420 A421 A422 A427 A428 A429 A46 A46X A480 A481 A482 A483 A484 A488 A490 A491 A492 A493 A498 A499 A810 A811 A812 A818 A819 A850 A852 A858 A86X A86 A870 A871 A872 A878 A879 A880 A881 A888 A89 A89X B001 B002 B003 B004 B005 B007 B008 B009 B010 B011 B012 B018 B019 B020 B021 B022 B023 B027 B028 B029 B07X B07 B080 B081 B082 B083 B084 B085 B088 B09X B150 B159 B160 B161 B162 B169 B170 B171 B172 B178 B179 B190 B199 B250 B251 B252 B258 B259 B270 B271 B278 B279 B300 B301 B302 B303 B308 B309 B330 B331 B332 B333 B334 B338 B340 B341 B342 B343 B344 B348 B349 B371 B372 B373 B374 B375 B376 B377 B378 B379 B440 B441 B442 B447 B448 B449 B450 B451 B452 B453 B457 B458 B459 B49X B59X B950 B951 B952 B953 B954 B955 B956 B957 B958 B960 B961 B962 B963 B964 B965 B966 B967 B968 B970 B971 B972 B973 B974 B975 B976 B977 B978 B99 B99X J200 J201 J202 J203 J204 J205 J206 J207 J208 J209 J22X J120 J121 J122 J123 J128 J129 J13 J14 J13X J14X J150 J151 J152 J153 J154 J155 J156 J157 J158 J159 J160 J168 J170 J171 J172 J173 J178 J180 J181 J182 J188 J189 J09 J100 J101 J108 J110 J111 J118 J850 J851 J852 J853 J860 J869 N10X N390 N300 N308 N309 N340 N151 N450 N459 N410 N412 N413 L00X L010 L011 L020 L021 L022 L023 L024 L028 L029 L030 L031 L032 L033 L038 L039 L040 L041 L042 L043 L048 L049 L050 L059 L080 L081 L088 L089 N700 N709 N710 N72X N730 N732 N733 N735 N760 N762 N764 N61X T814 G000 G001 G002 G003 G008 G009 G01X G020 G021 G028 G030 G038 G039 G040 G041 G042 G048 G049 G050 G051 G052 G058 G060 G061 G062 G07X G08X A851 M600 I330 I339 I300 I301 I308 I309 I400 I401 I408 I409 I514 I518 H700 K052 K113 J040 J041 J042 H600 H601 H603 H660 J010 J011 J012 J013 J014 J018 J019 J020 J028 J029 J030 J038 J039 M871 K102 M860 M861 M869 M000 M001 M002 M008 M009 K750 K610 K611 K612 K613 K614 K800 K803 K804 K810 K830 K630 K65 K65X
**Neutropenic sepsis**	*Defined where neutropenia & infection are recorded for the same admission.*
**Gastrointestinal disorder**	K521 K528 K529 A090 A099 R110 R111 R112 R11X R13X K590 K564 K121 K123 B370 K710 K711 K712 K716 K719 K720 K729 R17 R17X K221 K223 K251 K253 K255 K261 K262 K263 K265 K271 K273 K275 K281 K283 K285 K291 K293 K295 K631 K914 N321 N820 N822 N823 N824 K316 K603 K605 K604
**Cardiovascular**	
Arrhythmia	I440* I441* I442* I443* I444* I445* I446* I447* I471* I472* I480* I483* I484* I489* I48X* I450* I451* I452* I453* I454* I455* I456* I458* I459* I490* I491* I492* I493* I494* I495* I498* I499* R000 R001 R002 R008
Hypertension	I10* I10X* I110* I119* I120* I129* I130* I131* I132* I139* I150* I151* I152* I158* I159*
Angina	I200* I201* I208* I209*
Congestive cardiac failure	I500* I501* I509*
Cerebrovascular	I630* I631* I632* I633* I634* I635* I636* I638* I639* I600* I601* I602* I603* I604* I605* I606* I607* I608* I609* I64* I64X* I610* I611* I612* I613* I614* I615* I616* I618* I619* I620* I621* I629* I690* I691* I692* I693* I694* I698* G450* G451* G452* G453* G454* G458* G459* G460* G461* G462* G463* G464* G465* G466* G467* G468*
Other	I210 I211 I212 I213 I214 I219 I220 I221 I228 I229 I230 I231 I232 I233 I234 I235 I236 I238 I950 I951 I952 I958 I959 I260 I269 I313 I319 I427 I429 I740 I741 I742 I743 I744 I745 I748 I749 I822 I823 I828 I829 I800 I801 I802 I803 I808 I809

**Pain**	R100 R101 R102 R103 R104 M255 M540 M541 M542 M543 M544 M545<br>M546 M548 M549 R07 R07X R070 R071 R072 R073 R074 R520 R529 H920 K146 H571 M796
**Psychological disorder**	
Depression	F329*
Anxiety	F419*
Other	F320 F321 F322 F323 F328 F410 F411 F412 F413 F418
**Constitutional**	R530 R531 R538 R53X R64 R64X R630 R634 R638 E877 E860 E86X E861 E869 R600 R601 R609 R60X
**Neurological**	
Epilepsy	G400* G401* G402* G403* G404* G405* G406* G407* G408* G409* G410* G411* G412* G418* G419* R56* R560* R568*
Other	R55X R55 R42 R42X<br>G620 G628 G629 R200 R201 R202 R203 R208 R209 H910 H931 J385 G250 G251 G252 G253 G258 G259 G240 G254 G256 G711 G720 R270 R260 G430 G431 G432 G433 G438 G439 G440 G441 G442 G443 G444 G448 R51 R51X
**Metabolic/endocrine disorder**	E870 E871 E872 E873 E874 E875 E876 E878 E833 E835 E838 E839 E883 E834 R730 R739 E15 E15X E160 E161 E162 E032 E058 E064 E273 E231
**Renal disorder**	N170 N171 N172 N178 N179 N19X N19 N10 N10X N12X N12 N130 N131 N132 N133 N134 N135 N136 N137 N138 N139 N141 N142 N144 N158 N159 N280
**Line complications**	T825 T827 T828 T829 Z452 T800 T801 T802 T808 T809
**Dermatology & rheumatology**	
Gout	M100* M102* M104* M109*
Other	R21X R21 L270 L271 L298 L299 L51 L510 L511 L512 L518 L519 L539 R238 R239
**Respiratory disorder**	R05X R05 J80X J80 J81 J81X R060
**Bleeding**	R040 R310 R31X N938 N939 R042 J942 K625 I850 K920 K921 K922 K250 K252 K254 K256 K260 K262 K264 K266 K270 K272 K274 K276 K280 K282 K284 K286 K290 K292 K294 K296
**Ophthalmic disorder**	
Retinal disorder	H300* H301* H302* H308* H309* H310* H311* H313* H314* H318* H319* H330* H332* H335* H340* H341* H342* H348* H349* H350* H352* H353* H356* H357* H358* H359*
Blindness/visual impairment	H540* H541* H542* H543* H544* H545* H546* H549*
Visual disorder	H490* H491* H492* H493* H494* H498* H499* H500* H501* H502* H503* H504* H505* H506* H508* H509* H510* H511* H512* H518* H519*<br>H46X* H46* H470* H471* H472* H473* H474* H475* H476* H477*
Other	H320 H191 H192 H10 H100 H101 H102 H103 H105 H108 H109 H11 H111 H112 H113 B300 B301 B302 B303 B308 B309 H150 H151 H158 H159 H160 H161 H162 H163 H164 H168 H169 M350 H170 H171 H178 H179 H180 H181 H182 H183 H184 H186 H187 H188 H189 H200 H202 H208 H209 H210 H211 H212 H213 H214 H215 H218 H219 H263 H278 H279 H406 H531 H532 H533 H534 H535 H536 H538 H539<br>H000 H001 H010 H018 H019 H041 H042 H043 H020 H021 H050 H052 H058 H059 H578 H579<br>H431 H432 H433 H438 H439 H440 H441 H448 H449
**Drug reaction**	L500 T782 T783 T784 T886 T887 T451

*Codes excluded if present in the 12 months preceding treatment administration

Table A2: Baseline characteristics (inclusion/exclusion criteria) of patients in each adjuvant trastuzumab trial and associated routine data used to define trial eligibility within the cohort of women receiving adjuvant trastuzumab-based treatment for HER2-positive, early invasive breast cancer

Characteristics	Adjuvant Trastuzumab Trial						Data used to define trial eligibility (Yes/No) in routine care
	**B31** AC-P vs AC-PH	**BCIRG006** AC-T vs AC-TH	**FinHer** T/V-FEC vs T/VH-FEC	**HERA** Obs vs H	**N9831** AC-P vs AC-PH	**PACS-04** FEC/ET-Obs/H	
**Age (years)**	22-78	22-74	25-65	18-79	19-82	22-65	Age at diagnosis
**Sex**	Female	Female	Female	Female	Female	Female	Gender
**Performance status**		Karnofsky PS >80%	WHO PS 0/1				WHO performance status 0/1
**Hepatic function**	Adequate	Normal	Exclude if abnormal	Adequate	Adequate		No Liver comorbidity
**Renal function**	Adequate	Normal		Adequate	Adequate		No Renal comorbidity
**Cardiac function**	LVEF met or exceeded lower limit of normal	Normal.		Exclude if baseline LVEF <55%.	LVEF met or exceeded lower limit of normal	Exclude if LVEF <50%	No MI or CCF comorbidity
**Comorbidity**	Exclude if history of CCF/MI/ cardiomyopathy	Exclude if any cardiac disease or diabetes	Exclude if severe hypertension or any cardiac disease, history of CCF /MI	Exclude if history of documented CCF	Exclude if history of CCF /MI/ cardiomyopathy	Exclude if signs of CCF	
**Other unstable conditions incl. dementia.**		Exclude if history of dementia					No dementia comorbidity
**Hormone status**			PR-negative				PR status record
**HER2 status**	HER2-positive	HER2-positive	HER2-positive	HER2-positive	HER2-positive	HER2-positive	HER2 status record
**Tumour stage**		Exclude if T4		Exclude if T4		Exclude if T4 or greater	Tumour stage
**Nodal stage**	N+	N+, high risk N0 (tumour size≥2cm). Exclude if N2/3	N+, or N0 with tumour size≥2cm	N+, or N0 with tumour size>1cm	N+, high risk N0 (tumour size>2cm OR >1cm & HR-negative)	N+	N stage (& tumour size for high risk)
**Metastatic disease**	Exclude if evidence of metastatic disease	Exclude if M1	Exclude if distant metastases	Exclude if distant metastases	Exclude if evidence of metastatic disease	Exclude if suspected metastases	Stage 4 record
**Surgery**	Complete resection of primary tumour	Definitive surgery of the breast cancer	Breast surgery	Complete excision of the cancer	Complete resection of primary tumour	Cancer completely surgically removed	BCS/mastectomy record
**Other surgical procedure**	Axillary-node dissection	Axillary lymph node assessment	Axillary-node dissection or sentinel-node biopsy		Axillary-node dissection		AND/SNB record
**Prior**		No prior systemic therapy		No prior anti-HER2 therapy			Neoadjuvant chemo or HER2 therapy recorded
**Other unstable conditions incl. dementia.**		No history of dementia					Dementia comorbidity recorded

Gannon MR, *et al. BMJ Oncology* 2024; 2:e000081. doi: 10.1136/bmjonc-2023-000081

Table A3: Pre-specified conditions included in the assignment of Charlson Comorbidity Index

**Conditions**			
Myocardial infarction	Dementia	Diabetes mellitus	Metastatic solid tumour
Congestive cardiac failure	Chronic pulmonary disease	Hemiplegia or paraplegia	AIDS/HIV infection
Peripheral vascular disease	Rheumatological disease	Renal disease	
Cerebrovascular disease	Liver disease	Any malignancy	

Gannon MR, *et al. BMJ Oncology* 2024; 2:e000081. doi: 10.1136/bmjonc-2023-000081

Table A4: Comparison of patient, tumour and treatment characteristics among women receiving adjuvant treatment for early invasive breast cancer, by HER2 status

Characteristic		Total N = 18664		HER2-positive adjuvant trastuzumab N = 5087		HER2-negative adjuvant chemotherapy N = 13557	
		N	%	N	%	N	%
**Age group**	50-59 yrs	8726	46.8%	2153	42.3%	6573	48.4%
	60-69 yrs	6943	37.2%	1906	37.5%	5037	37.1%
	70-79 yrs	2859	15.3%	941	18.5%	1918	14.1%
	80+ yrs	136	0.7%	87	1.7%	49	0.4%
**IMD**	1 - Most deprived	2957	15.8%	772	15.2%	2185	16.1%
	2	3296	17.7%	852	16.7%	2444	18.0%
	3	3935	21.1%	1088	21.4%	2847	21.0%
	4	4206	22.5%	1162	22.8%	3044	22.4%
	5 - Least deprived	4270	22.9%	1213	23.8%	3057	22.5%
**Charlson Comorbidity Index**	**0**	17100	91.6%	4609	90.6%	12491	92.0%
	1	1241	6.6%	381	7.5%	860	6.3%
	2+	323	1.7%	97	1.9%	226	1.7%
**SCARF Index**	Fit	16083	86.2%	4358	85.7%	11725	86.4%
	Mild frailty	1759	9.4%	474	9.3%	1285	9.5%
	Moderate - severe frailty	822	4.4%	255	5.0%	567	4.2%
**Stage grouping**	1	5455	29.2%	2365	46.5%	3090	22.8%
	2	10598	56.8%	2361	46.4%	8237	60.7%
	3A	2611	14.0%	361	7.1%	2250	16.6%
**Grade of disease**	G1	527	2.8%	98	1.9%	429	3.2%
	G2	7419	39.8%	1868	36.7%	5551	40.9%
	G3	10718	57.4%	3121	61.4%	7597	56.0%
**Tumour stage**	T1	8092	43.4%	2863	56.3%	5229	38.5%
	T2	9232	49.5%	2056	40.4%	7176	52.9%
	T3	1340	7.2%	168	3.3%	1172	8.6%
**Nodal stage**	N0	9487	50.8%	3499	68.8%	5988	44.1%
	N1	7187	38.5%	1291	25.4%	5896	43.4%
	N2	1990	10.7%	297	5.8%	1693	12.5%
**Positive hormone-receptor status**	Yes	12864	68.9%	3504	68.9%	9360	68.9%
	No/Unknown	5800	31.1%	1583	31.1%	4217	31.1%
**Surgery type**	BCS	12552	67.3%	3477	68.4%	9075	66.8%
	Mastectomy	6112	32.7%	1610	31.6%	4502	33.2%
**Radiotherapy reported/setting**	No	2777	14.9%	1055	20.7%	1722	12.7%
	Yes – before treatment	335	1.8%	301	5.9%	34	0.3%
	Yes – during treatment	2958	15.8%	2958	58.1%	0	0.0%
	Yes – after treatment	12594	67.5%	773	15.2%	11821	87.1%
**Hormone therapy prescribed (Y)**		12825	68.7%	3508	69.0%	9317	68.6%
**Chemotherapy reported (Y)**		18592	99.6%	5015	98.6%	13577	100.0%
	Anthracycline only (Y)	5079	27.3%	911	18.2%	4168	30.7%
	Taxane only (Y)	3463	18.6%	2070	41.3%	1393	10.3%
	Docetaxel (Y)	9626	51.8%	2484	49.5%	7142	52.6%
	Paclitaxel (Y)	4046	21.8%	1639	32.7%	2407	17.7%
	Anthracycline and taxane (Y)	9904	53.3%	1988	39.6%	7916	58.3%

Note: Anthracyclines = doxorubicin, epirubicin, mitoxantrone recorded in SACT.
Taxanes = docetaxel, cabazitaxel, paclitaxel, nab-paclitaxel recorded in SACT.

Gannon MR, *et al. BMJ Oncology* 2024; 2:e000081. doi: 10.1136/bmjonc-2023-000081

Table A5: Frequency of severe acute toxicity events (SATE), overall / by individual SATE type, among women receiving adjuvant trastuzumab-based treatment for HER2-positive, early invasive breast cancer, by sequential or concurrent use of chemotherapy *(ordered by the most frequently recorded; only individual SATE with >5% presented)*

Event	Total N = 5087		Sequential chemotherapy N = 1160		Concurrent chemotherapy N = 3927	
	N	%	N	%	N	%
**Any**	1670	32.8%	402	34.7%	1268	32.3%
**Haematological**	774	15.2%	213	18.4%	561	14.3%
Neutropenia	*714*	*14.0%*	*205*	*17.7%*	*509*	*13.0%*
Anaemia	*103*	*2.0%*	*26*	*2.2%*	*77*	*2.0%*
Thrombocytopenia	*15*	*0.3%*	*2*	*0.2%*	*13*	*0.3%*
**Infection**	773	15.2%	151	13.0%	622	15.8%
**Neutropenic sepsis**	659	13.0%	188	16.2%	471	12.0%
**Gastrointestinal**	507	10.0%	100	8.6%	407	10.4%
**Cardiovascular**	346	6.8%	102	8.8%	244	6.2%
Arrhythmia	*106*	*2.1%*	*29*	*2.5%*	*77*	*2.0%*
Hypertension	*73*	*1.4%*	*16*	*1.4%*	*57*	*1.5%*
Angina	*7*	*0.1%*	*0*	*0.0%*	*7*	*0.2%*
Congestive cardiac failure	*25*	*0.5%*	*7*	*0.6%*	*18*	*0.5%*
Cerebrovascular	*14*	*0.3%*	*7*	*0.6%*	*7*	*0.2%*
Other	*175*	*3.4%*	*64*	*5.5%*	*111*	*2.8%*

Gannon MR, *et al. BMJ Oncology* 2024; 2:e000081. doi: 10.1136/bmjonc-2023-000081

Table A6: Frequency of severe acute toxicity events (SATE), overall / by individual SATE type, among women receiving adjuvant treatment for early invasive breast cancer, by HER2 status
(ordered by the most frequently recorded; only individual SATE with >5% presented)

Event	Total N = 18664		HER2-positive adjuvant trastuzumab N = 5087		HER2-negative adjuvant chemotherapy N = 13557	
	N	%	N	%	N	%
**Any**	6784	36.3%	1670	32.8%	5115	37.7%
**Haematological**	3430	18.4%	774	15.2%	2656	19.6%
Neutropenia	*3212*	*17.2%*	*714*	*14.0%*	*2498*	*18.4%*
Anaemia	*415*	*2.2%*	*103*	*2.0%*	*312*	*2.3%*
Thrombocytopenia	*98*	*0.5%*	*15*	*0.3%*	*83*	*0.6%*
**Infection**	3052	16.4%	773	15.2%	2279	16.8%
**Neutropenic sepsis**	2918	15.6%	659	13.0%	2259	16.6%
**Gastrointestinal disorder**	2171	11.6%	507	10.0%	1668	12.3%
**Cardiovascular**	1505	8.1%	346	6.8%	1159	8.5%
Arrhythmia	*492*	*2.6%*	*106*	*2.1%*	*386*	*2.8%*
Hypertension	*309*	*1.7%*	*73*	*1.4%*	*236*	*1.7%*
Angina	*42*	*0.2%*	*7*	*0.1%*	*35*	*0.3%*
Congestive cardiac failure	*109*	*0.6%*	*25*	*0.5%*	*84*	*0.6%*
Cerebrovascular	*66*	*0.4%*	*14*	*0.3%*	*52*	*0.4%*
Other	*751*	*4.0%*	*175*	*3.4%*	*576*	*4.2%*
**Pain**	954	5.1%	220	4.3%	734	5.4%

Gannon MR, *et al. BMJ Oncology* 2024; 2:e000081. doi: 10.1136/bmjonc-2023-000081

Table A7: Frequency of severe acute toxicity events (SATE), overall / by individual SATE type, among women receiving adjuvant trastuzumab-based treatment for HER2-positive early invasive breast cancer, by comorbidity burden *(ordered by the most frequently recorded; only individual SATE with >5% presented)*

Event	CCI 0 N = 4609		CCI 1 N = 387		CCI 2+ N = 97	
	N	%	N	%	N	%
**Any**	1461	31.7%	161	42.3%	48	49.5%
**Haematological**	694	15.1%	64	16.8%	16	16.5%
Neutropenia	645	14.0%	56	14.7%	13	13.4%
Anaemia	84	1.8%	15	3.9%	4	4.1%
Thrombocytopenia	11	0.2%	3	0.8%	1	1.0%
**Infection**	666	14.4%	79	20.7%	28	28.9%
**Neutropenic sepsis**	596	12.9%	50	13.1%	13	13.4%
**Gastrointestinal disorder**	433	9.4%	55	14.4%	19	19.6%
**Cardiovascular**	291	6.3%	38	10.0%	17	17.5%
Arrhythmia	90	2.0%	11	2.9%	5	5.2%
Hypertension	64	1.4%	8	2.1%	1	1.0%
Angina	1	0.0%	5	1.3%	1	1.0%
Congestive cardiac failure	15	0.3%	6	1.6%	4	4.1%
Cerebrovascular	12	0.3%	2	0.5%	0	0.0%
Other	152	3.3%	14	3.7%	9	9.3%
**Pain**	182	3.9%	28	7.3%	10	10.3%
**Psychological disorder**	102	2.2%	12	3.1%	3	3.1%
Depression	59	1.3%	8	2.1%	3	3.1%
Anxiety	41	0.9%	2	0.5%	0	0.0%
Other	19	0.4%	4	1.0%	0	0.0%
**Constitutional**	128	2.8%	25	6.6%	10	10.3%
**Neurological**	125	2.7%	18	4.7%	6	6.2%
Epilepsy	7	0.2%	1	0.3%	0	0.0%
Other	121	2.6%	17	4.5%	6	6.2%
**Metabolic/endocrine disorder**	104	2.3%	23	6.0%	7	7.2%
**Renal disorder**	64	1.4%	16	4.2%	11	11.3%

Key: CCI = Charlson Comorbidity Index.

Table A8: Distribution of patient, tumour and treatment characteristics among women receiving adjuvant trastuzumab-based treatment for HER2-positive early invasive breast cancer, and associated odds of any severe acute toxicity events (SATE) or cardiovascular SATE.

Characteristic		All N	Any SATE N (%)	Any SATE OR (95% CI)	Any SATE Overall p-value	Cardiovascular SATE N (%)	Cardiovascular SATE OR (95% CI)	Cardiovascular SATE Overall p-value
**Total**		5087	1670 (32.8%)	-		346 (6.8%)	-	
**Age group**	50-59 years	2153	710 (33.0%)	1.00	0.615	132 (6.1%)	1.00	0.024
	60-69 years	1906	625 (32.8%)	1.00 (0.87-1.14)		125 (6.6%)	1.04 (0.80-1.35)	
	70-79 years	1028	335 (32.6%)	1.08 (0.91-1.28)		89 (8.7%)	1.46 (1.09-1.95)	
**IMD**	1 - Most deprived	772	302 (39.1%)	1.38 (1.13-1.69)	0.012	68 (8.8%)	1.53 (1.07-2.2)	0.077
	4	852	290 (34.0%)	1.12 (0.93-1.37)		63 (7.4%)	1.26 (0.88-1.82)	
	2	1088	349 (32.1%)	1.05 (0.87-1.26)		62 (5.7%)	1.01 (0.71-1.45)	
	3	1162	359 (30.9%)	1.01 (0.84-1.20)		87 (7.5%)	1.35 (0.97-1.89)	
	5 - Least deprived	1213	370 (30.5%)	1.00		66 (5.4%)	1.00	
**CCI**	0	4609	1461 (31.7%)	1.00	0.938	291 (6.3%)	1.00	0.680
	1	381	161 (42.3%)	1.08 (0.68-1.70)		38 (10%)	0.69 (0.29-1.60)	
	2+	97	48 (49.5%)	1.05 (0.45-2.44)		17 (17.5%)	0.55 (0.13-2.44)	
**History of MI**	No	5070	1660 (32.7%)	1.00	0.091	340 (6.7%)	1.00	0.002
	Yes	17	10 (58.8%)	2.53 (0.86-7.43)		6 (35.3%)	7.84 (2.17-28.34)	
**History of CCF**	No	5070	1662 (32.8%)	No crude association		343 (6.8%)	1.00	0.330
	Yes	17	8 (47.1%)	-		3 (17.6%)	2.16 (0.46-10.23)	
**History of diabetes**	No	4925	1597 (32.4%)	1.00	0.575	328 (6.7%)	1.00	0.278
	Yes	162	73 (45.1%)	1.16 (0.70-1.91)		18 (11.1%)	1.62 (0.68-3.88)	
**History of liver disease**	No	5055	1652 (32.7%)	1.00	0.150	339 (6.7%)	1.00	0.009
	Yes	32	18 (56.3%)	1.82 (0.81-4.11)		7 (21.9%)	1.62 (0.68-3.88)	
**History of CPD**	No	4858	1570 (32.3%)	1.00	0.418	315 (6.5%)	1.00	0.024
	Yes	229	100 (43.7%)	1.23 (0.75-2.02)		31 (13.5%)	2.67 (1.14-6.25)	
**History of renal disease**	No	5049	1651 (32.7%)	1.00	0.204	343 (6.8%)	No crude association	
	Yes	38	19 (50%)	1.65 (0.76-3.58)		3 (7.9%)	-	
**SCARF Index**	Fit	4358	1364 (31.3%)	1.00	0.005	278 (6.4%)	1.00	0.490
	Mild frailty	474	193 (40.7%)	1.42 (1.13-1.79)		35 (7.4%)	0.92 (0.59-1.42)	
	Moderate - severe frailty	255	113 (44.3%)	1.47 (1.03-2.10)		33 (12.9%)	1.30 (0.73-2.34)	
**Anthracycline chemotherapy**	No	2116	593 (28%)	1.00	<0.0001	135 (6.4%)	No crude association	
	Yes	2899	1065 (36.7%)	1.55 (1.36-1.78)		207 (7.1%)	-	
	No chemotherapy	72	12 (16.7%)	0.47 (0.24-0.89)		4 (5.6%)	-	
**Sequential chemotherapy**	No	3916	1263 (32.3%)	No crude association		242 (6.2%)	1.00	0.003
	Yes	1099	395 (35.9%)	-		100 (9.1%)	1.54 (1.19-1.98)	
	No chemotherapy	72	12 (16.7%)	-		4 (5.6%)	0.69 (0.24-2.03)	

Key: IMD = Index of Multiple Deprivation; CCI = Charlson Comorbidity Index; MI = myocardial infarction; CCF = congestive cardiac failure; CPD = Chronic pulmonary disease; SCARF = Secondary Care Administrative Records Frailty; chemo = chemotherapy.
Note: ORs adjusted for other factors and year of diagnosis.

Table A9: Relationship between adjuvant trastuzumab for HER2-positive early invasive breast cancer and any severe acute toxicity events (SATE).

	Total	50-59 years	60-69 years	70-79 years	80+ years
**Cycle-based analysis**	**N = 5087**	**N = 2153**	**N = 1906**	**N = 941**	**N = 87**
**Experienced SATE AND**					
No further cycles (discontinued)	67 (1.3%)	21 (1.0%)	14 (0.7%)	25 (2.7%)	7 (8.0%)
Delay/break before next cycle	402 (7.9%)	147 (6.8%)	157 (8.2%)	91 (9.7%)	7 (8.0%)
Delay/break after any subsequent cycle	25 (0.5%)	9 (0.4%)	12 (0.6%)	4 (0.4%)	0 (0.0%)
No delay/break before end of treatment	4 (0.1%)	1 (0.0%)	1 (0.1%)	1 (0.1%)	1 (1.1%)
**No SATE recorded AND**					
Delay/break before next cycle	3323 (65.3%)	1414 (65.7%)	1258 (66.0%)	590 (62.7%)	61 (70.1%)
**Patient-based analysis**					
**Experienced SATE AND**					
Treatment completed	586 (11.5%)	256 (11.9%)	221 (11.6%)	102 (10.8%)	7 (8.0%)
Treatment included delays/breaks	866 (17.0%)	344 (16.0%)	330 (17.3%)	176 (18.7%)	16 (18.4%)
Treatment continued* after SATE but stopped early	1 (0.0%)	1 (0.0%)	0 (0.0%)	0 (0.0%)	0 (0.0%)
Treatment stopped after SATE	67 (1.3%)	21 (1.0%)	14 (0.7%)	25 (2.7%)	7 (8.0%)
**No SATE recorded AND**					
Treatment completed	1823 (35.8%)	783 (36.4%)	689 (36.1%)	324 (34.4%)	27 (31.0%)
Treatment included delays/breaks	2629 (51.7%)	1132 (52.6%)	989 (51.9%)	461 (49.0%)	47 (54.0%)
Treatment discontinued	2107 (41.4%)	897 (41.7%)	792 (41.6%)	379 (40.3%)	39 (44.8%)

*With no delays/breaks

Gannon MR, *et al. BMJ Oncology* 2024; 2:e000081. doi: 10.1136/bmjonc-2023-000081

Table A10: Distribution of patient, tumour and treatment characteristics among women receiving adjuvant trastuzumab for HER2-positive early invasive breast cancer, diagnosed in NHS trusts in England between January 2014 and December 2019, by trial eligibility.

		**Total N = 5087**		**Not trial eligible N = 3274**		**Trial eligible N = 1813**	
**Characteristic**		N	%	N	Row %	N	Row %
**Age group**	50-59 years	2153	42.3%	1365	63.4%	788	36.6%
	60-69 years	1906	37.5%	1262	66.2%	644	33.8%
	70-79 years	941	18.5%	585	62.2%	356	37.8%
	80+ years	87	1.7%	62	71.3%	25	28.7%
**IMD**	1 - Most deprived	772	15.2%	477	61.8%	295	38.2%
	2	852	16.7%	529	62.1%	323	37.9%
	3	1088	21.4%	702	64.5%	386	35.5%
	4	1162	22.8%	756	65.1%	406	34.9%
	5 - Least deprived	1213	23.8%	810	66.8%	403	33.2%
**Charlson Comorbidity Index**	**0**	4609	90.6%	2925	63.5%	1684	36.5%
	1	381	7.5%	268	70.3%	113	29.7%
	2+	97	1.9%	81	83.5%	16	16.5%
**SCARF Index**	Fit	4358	85.7%	2764	63.4%	1594	36.6%
	Mild frailty	474	9.3%	316	66.7%	158	33.3%
	Moderate - severe frailty	255	5.0%	194	76.1%	61	23.9%
**Stage grouping**	1	2365	46.5%	1973	83.4%	392	16.6%
	2	2361	46.4%	1258	53.3%	1103	46.7%
	3A	361	7.1%	43	11.9%	318	88.1%
**Grade of disease**	G1	98	1.9%	74	75.5%	24	24.5%
	G2	1868	36.7%	1260	67.5%	608	32.5%
	G3	3121	61.4%	1940	62.2%	1181	37.8%
**Tumour stage**	T1	2863	56.3%	2110	73.7%	753	26.3%
	T2	2056	40.4%	1104	53.7%	952	46.3%
	T3	168	3.3%	60	35.7%	108	64.3%
**Nodal stage**	N0	3499	68.8%	2790	79.7%	709	20.3%
	N1	1291	25.4%	456	35.3%	835	64.7%
	N2	297	5.8%	28	9.4%	269	90.6%
**Positive hormone-receptor status**	Yes	3504	68.9%	2306	65.8%	1198	34.2%
	No/Unknown	1583	31.1%	968	61.1%	615	38.9%
**Surgery type**	BCS	3477	68.4%	2414	69.4%	1063	30.6%
	Mastectomy	1610	31.6%	860	53.4%	750	46.6%
**Radiotherapy reported/setting**	No	1055	20.7%	725	68.7%	330	31.3%
	Yes – before treatment	301	5.9%	76	25.2%	225	74.8%
	Yes – during treatment	2958	58.1%	1951	66.0%	1007	34.0%
	Yes – after treatment	773	15.2%	522	67.5%	251	32.5%
**Hormone therapy prescribed**	No	1579	31.0%	981	62.1%	598	37.9%
	Yes	3508	69.0%	2293	65.4%	1215	34.6%
**Chemotherapy reported**	No	72	1.4%	54	75.0%	18	25.0%
	Yes	5015	98.6%	3220	64.2%	1795	35.8%
	Anthracycline only – No	4104	81.8%	2956	72.0%	1148	28.0%
	Anthracycline only – Yes	911	18.2%	264	29.0%	647	71.0%
	Taxane only – No	2945	58.7%	1519	51.6%	1426	48.4%
	Taxane only – Yes	2070	41.3%	1701	82.2%	369	17.8%
	Docetaxel – No	2531	50.5%	1573	62.1%	958	37.9%
	Docetaxel – Yes	2484	49.5%	1647	66.3%	837	33.7%
	Paclitaxel – No	3376	67.3%	1899	56.3%	1477	43.8%
	Paclitaxel – Yes	1639	32.7%	1321	80.6%	318	19.4%
	Anthracycline and taxane – No	3027	60.4%	1989	65.7%	1038	34.3%
	Anthracycline and taxane – Yes	1988	39.6%	1231	61.9%	757	38.1%

Note: Anthracyclines = doxorubicin, epirubicin, mitoxantrone recorded in SACT.
Taxanes = docetaxel, cabazitaxel, paclitaxel, nab-paclitaxel recorded in SACT.
